# Enhanced thermodynamic, pharmacokinetic and theranostic properties of polymeric micelles via hydrophobic core-clustering of superparamagnetic iron oxide nanoparticles

**DOI:** 10.1186/s40824-022-00255-9

**Published:** 2022-03-07

**Authors:** Yixin Jiang, Junghan Lee, Jin-Myung Seo, Enkhzaya Davaa, Kyung-Ju Shin, Su-Geun Yang

**Affiliations:** 1 Department of Biomedical Science, BK21 FOUR Program in Biomedical Science and Engineering, Inha University College of Medicine, B-308, Chungsuk Bldg, 366, Seohae-Daero, Jung-Gu, Incheon, 22212 Republic of Korea; 2 Inha Institute of Aerospace Medicine, Inha University College of Medicine, Incheon, 22332 Republic of Korea

**Keywords:** Polymeric micelles, Superparamagnetic iron oxide, Critical micelle concentration, Theranostic nanoparticle, Micelle stability, SPIO clustering

## Abstract

**Background:**

Superparamagnetic iron oxide nanoparticles (SPIO) have been applied for decades to design theranostic polymeric micelles for targeted cancer therapy and diagnostic MR imaging. However, the effects of SPIO on the physicochemical, and biological properties of polymeric micelles have not yet been fully elucidated. Therefore, we investigated potential effect of SPIO on the physical and biological properties of theranostic polymeric micelles using representative cancer drug (doxorubicin; Doxo) and polymer carrier (i.e., poly (ethylene glycol)-co-poly(D,L-lactide), PEG-PLA).

**Methods:**

SPIO were synthesized from Fe(acetyl acetonate)_3_ in an aryl ether. SPIO and Doxo were loaded into the polymeric micelles by a solvent-evaporation method. We observed the effect of SPIO-clustering on drug loading, micelle size, thermodynamic stability, and theranostic property of PEG-PLA polymeric micelles. In addition, cellular uptake behaviors, pharmacokinetic and biodistribution study were performed.

**Results:**

SPIO formed hydrophobic geometric cavity in the micelle core and significantly affected the integrity of micelles in terms of micelle size, Doxo loading, critical micelle concentration (CMC) and in vitro dissociation. In vivo pharmacokinetic studies also showed the enhanced Area Under Curve (AUC) and elongated the half-life of Doxo.

**Conclusions:**

Clustered SPIO in micelles largely affects not only MR imaging properties but also biological and physical properties of polymeric micelles.

**Supplementary Information:**

The online version contains supplementary material available at 10.1186/s40824-022-00255-9.

## Background

Multifunctional nanomedicine that integrates diagnostic and therapeutic functions has received substantial attention as the next generation of medicine [1–9]. In one system, multifunctional nanomedicine has the potential to provide molecular diagnosis, targeted therapy, and simultaneous treatment and monitoring of therapeutic efficacy, unlike traditional small molecular contrast agents or drugs [3, 10–13]. The ideal theranostic nanomedicine should provide high drug loading densities, drug-carrier bio-compatibility, responsive release mechanism to improve drug delivery efficiency, and imaging sensitivity to pre-validate and monitor therapy. One of the major challenges for the development of nanomedicine is the incorporation of multiple functional components (i.e., therapeutic drugs, imaging agents, and polymer carriers) into one nanocomposite system within a small size confinement (e.g., < 100 nm). Despite the explosive development of many multifunctional nanomedicine platforms, the interactions between each component and the influence of one component over the performance of the others have not been systematically investigated. Understanding of the material interactions at the nanoscale is critically important for the successful development and implementation of theranostic nanomaterials in medicine.

**Fig. 1:**
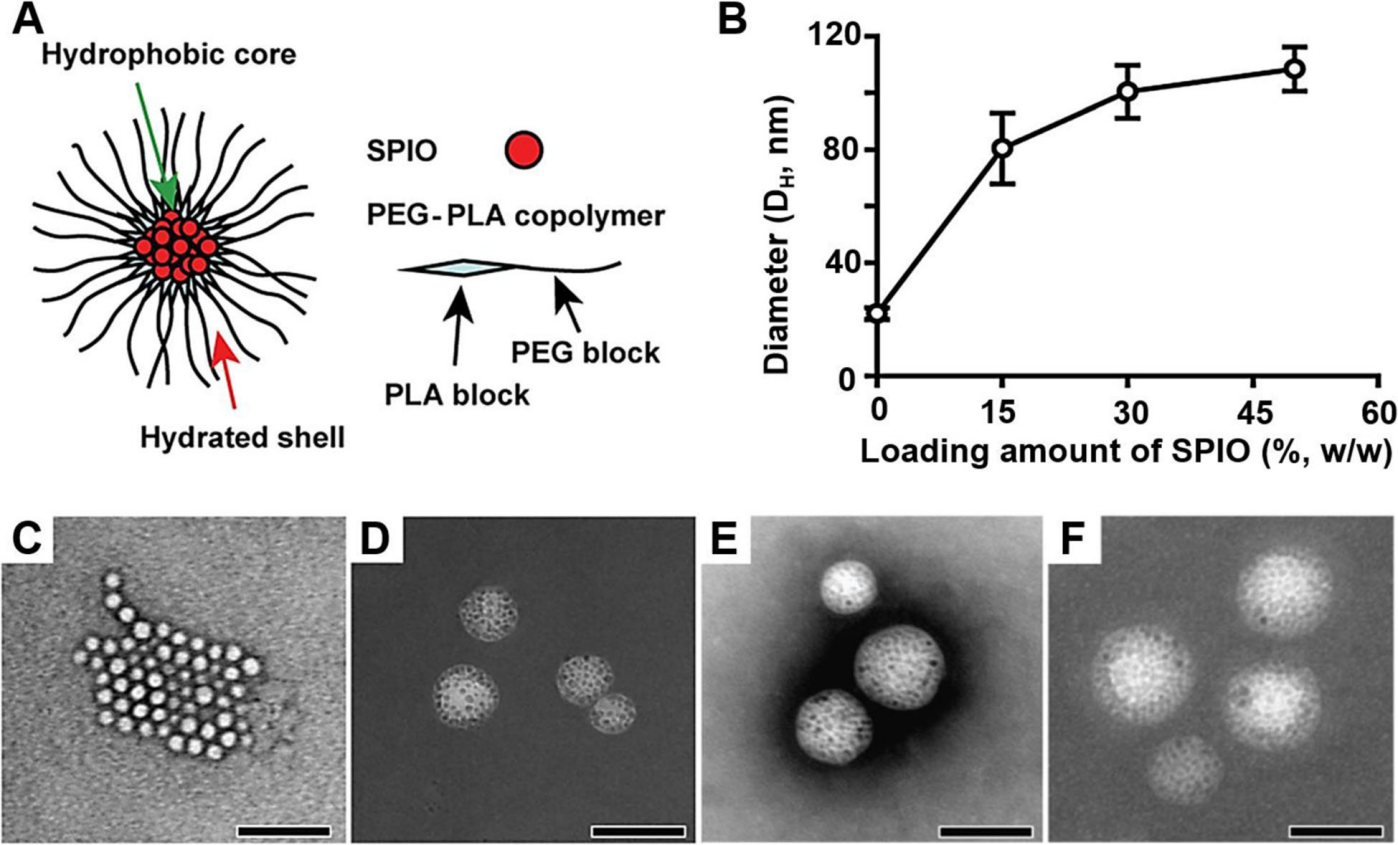
Effect of SPIO-clustering and loading on structure of PEG-PLA polymeric micelles. **A**) Structure of SPIO-loaded PEG-PLA micelles. (**B**) Hydrodynamic diameter of PEG-PLA polymeric micelles with 0, 15, 30, and 50% Doxo-loading contents of SPIO. (**C**-**F**) TEM images of the micelles with 0, 15, 30, and 50% Doxo-loading contents, Scale bar = 100 nm

Theranostic polymeric micelles have been studied for cancer molecular imaging and targeted drug delivery applications for decades [8, 14, 15]. An anticancer drug, doxorubicin (Doxo) and an imaging agent, superparamagnetic iron oxide nanoparticles (SPIO) were co-loaded inside the micelle core (Fig. 1A) and the micelle surface was further functionalized with a peptide that targets cell surface markers over-expressed in the tumor vasculature. Previous studies showed that clustering of hydrophobic SPIO in the hydrophobic micelle core resulted in dramatically increased T_2_ relaxivity, which allowed for the subsequent cancer-specific imaging of α_v_β_3_ integrins in solid tumors in vivo by magnetic resonance imaging (MRI) [1, 16–20].

In this paper, we evaluated the dynamics of interactions between the imaging agent (i.e., SPIO), small molecular drug (i.e., Doxo) and polymer carrier (i.e., poly (ethylene glycol)-co-poly(D, L-lactide), PEG-PLA). The effect of SPIO encapsulation on loading density of Doxo inside polymeric micelles and AUC during the administration of SPIO-micelles were investigated. Furthermore, we hypothesized SPIO-loaded micelles would correlate well with increased thermodynamic as well as kinetic stabilities in aqueous solution over SPIO-free micelles. It was demonstrated the physico-chemical insights and structure-property relationships for the further optimization of multifunctional micellar nanomedicine.

## Methods

### Materials

Poly(ethylene glycol)-poly(D,L-lactide) (PEG-PLA, PEG and PLA M.W. = 5 kD) was synthesized following the reported method [21]. The molecular weight and polydispersity of PEG-PLA were also characterized by gel permeation chromatography and ^1^H-NMR. Doxorubicin-HCl for injection (2 mg/mL) was purchased from Bedford Laboratories (Adriamycin®, Bedford, OH), and treated with triethylamine to obtain the hydrophobic Doxo. Phenyl ether (99%), oleic acid (99%), benzyl ether (99%), oleylamine (> 70%), tetrahydrofuran (THF), 1,2-hexadecanediol (97%), hexane, iron(III) acetylacetonate and dimethyl sulfoxide (DMSO) were purchased from Sigma-Aldrich Chemical Co. (Saint Louis, MO). 1,2-Distearoyl-sn-glycero-3-phosphoethanolamine-N-[methoxy (polyethylene glycol)-5000] (ammonium salt) (DSPE-PEG) was ordered from Avanti Polar Lipids, Inc. (Alabaster, Al). All organic solvents were used with analytical grade.

### Preparation of Doxo-SPIO-micelles

Doxo-SPIO-micelles were synthesized based on the published procedure [17, 22]. In short, highly crystalline and monodisperse SPIO were synthesized from Fe(acetyl acetonate)_3_ in an aryl ether [22]. PEG-PLA was used as surfactant molecules to encapsulate SPIO and Doxo for the micelles. Subsequently, SPIO with 7 nm diameter and Doxo were loaded into the polymeric micelles by a solvent-evaporation method [17]. SPIO and Doxo were dissolved in 800 μl of THF and 200 μl of DMSO. Then, THF and DMSO were mixed together, and this mixture was added into water (3 mL) under sonication (LH700S, ULSSO HITECH Co.). After the sonication, the solution was shaken overnight to allow the organic solvent to evaporate. Finally, Doxo-SPIO-micelles were purified by Millipore centrifuge filtration (MW cut off: 30kD). The weight ratios (mg) of PEG-PLA:SPIO:Doxo were varied over three values: 10:2.5:2; 10:5:2; and 10:10:2.

### Characterization of Doxo-SPIO-micelles

#### Hydrodynamic diameter

The hydrodynamic diameter (D_H_) of the Doxo-SPIO-micelles was estimated by dynamic light scattering method (DLS, Nano-ZS90, Malvern Panalytical Co. United Kingdom). Doxo-SPIO-micelles, made with different ratios of PEG-PLA:SPIO:Doxo (10:10:2, 10:5:2, 10:2.5:2), were introduced to DLS.

#### Transmission electron microscopy (TEM) imaging

To confirm the size and morphology of Doxo-SPIO-micelles, transmission electron microscopy (TEM) images for nanoparticles were obtained using an FE-TEM (JEM 2100F, JEOL Co. Japan). For Doxo-SPIO-micelles, formvar coated-copper grids were glow discharged using a vacuum coating unit. Each sample solution was dropped on to the glow discharged grid. After 2 min of standing, removal excess solution was removed by blotting the grid against a filter paper. Negative staining (dark field) of sample was done by additional dropping of 2% phosphotungstic acid (PTA) solution to the grid. For SPIO, TEM samples were prepared by allowing a small drop of SPIO suspension in hexane to dry on carbon-coated copper grids without negative staining. All TEM images were obtained (120 kV).

#### Determination of drug loading content (DLC)

The DLC of the micelles was measured by UV-VIS analysis. At first, the solid micelle samples were obtained by frozen dry from micelle solutions. Next the dried samples were weighed and re-dissolved in a mixture of chloroform and DMSO (1:1, v/v) re-dissolved in a mixture of chloroform and DMSO (1:1, v/v) under bath-sonication for 30 min. The suspending SPIO were centrifuged down, and upper layer of solution was transferred to UV-VIS spectrometer (Spectra Max Plus 384 Microplate Reader, Molecular Devices, USA). The absorbance at 480 nm was measured to determine the Doxo content in the solution with a previously established calibration curve. The weight% of Doxo, entrapped in the core of micelle, was calculated from the dried weight of Doxo-SPIO-micelles and the amount of Doxo incorporated.

### Estimation of thermodynamic properties of Doxo-SPIO-micelles

#### Critical micelle concentration (CMC) determination

The CMC of the polymer was calculated to investigate the effect of SPIO on the micelle stability. Following the reported method, a solution of pyrene in dichloromethane was dried by a stream of nitrogen [23]. An aqueous solution of micelles was subsequently added to the dried film followed by gentle shaking for 72 h at 37 °C. The final concentration of pyrene was 2.0 × 10^− 8^ mol/L while that of PEG-PLA was varied from 0.1 to 100 μg/mL. The CMC was calculated by plotting the fluorescence intensity of pyrene (λ_em_ = 390 nm, λ_ex_ = 333 nm) as a function of PEG-PLA concentration.

#### Evaluation of dissociation kinetics of Doxo-SPIO-micelles

A dissociation of micelles at a concentration below the CMC (100 μg/mL and 8 μg/mL, respectively) was also evaluated. SPIO-loaded micelles and SPIO-free micelles encapsulating pyrene were prepared in HEPES buffer pH 7.4 at the polymer concentration of 1.5 mg/mL according to the previously reported method. After micelle formation, the micelle solution was diluted, and the fluorescent intensity (λ_ex_ = 333, λ_em_ = 390) was recorded at 37 °C over time.

#### Estimation of thermodynamic fluidity of micelle core

In this study, we uploaded 1,3-di (1,1′-pyrenyl) propane (DPP) dye in the micelle core and estimated the molecular state of DPP (i.e., excimer formation) to examine the microenvironmental fluidity of the micelle core. DPP was dissolved in chloroform, dried, and reconstituted with ethyl acetate. Then DPP was mixed with the SPIO-loaded or SPIO-free micelles for 72 h under N_2_ gas. The final concentration of DPP was 1.8 × 10^− 7^ M, while the polymer concentration was 100 μg/mL, above the CMC. The emission intensity of DPP excimer at 478 nm (I_e_) and monomer at 377 nm (I_m_) was measured as a function of temperature (λ_ex_ = 333 nm).

#### In vitro drug release study

Doxo-SPIO-micelles were transferred into dialysis tubes (MW cut-off: 50,000 Da, Spectrum Laboratories, USA). The tubes were immersed in 25 mL PBS (pH 7.4) or acetate buffered saline (pH 5.0) solutions. The release of Doxo from micelles was tested under mechanical shaking (100 rpm/min) at 37 °C. Removal buffered solution outside the dialysis bag was estimated the released amount of Doxo and replaced with fresh buffer solution during selected time intervals. Doxo concentration was calculated based on the fluorescence intensity (ex; 470 nm, em; 590 nm) with a previously established calibration curve. The error bars were obtained from triplicate samples.

### Estimation of MR imaging properties of Doxo-SPIO-micelles

#### Estimation of magnetization properties of Doxo-SPIO-micelles

The magnetization of Doxo-SPIO-micelles was measured to understand the MR properties. Samples were prepared by pipetting 5 μl of solution containing Doxo-SPIO-loaded micelles into a glass cell and attached to the probe with a small amount of silicon grease. To investigate magnetization were performed by using an alternating gradient magnetometer (AGM, Princenton Measurements) at room temperature and up to 1.4 T.

#### Estimation of T_2_ relaxivity of Doxo-SPIO-micelles

MR sensitivity of Doxo-SPIO-micelles was measured at 0.55 T (23.4 MHz) on a Resonance Maran Ultra scanner at 37 °C (Oxford instruments, UK). The measurement of T_2_ relaxation rates (1/T_2_, s^− 1^) were via using a CPMG (Carr Purcell Meiboom Gill) pulse sequence. T_1_ relaxation rates (1/T_1_, s^− 1^) were measured using the INVREC pulse sequence with the repetition time (TE) =5 × T_1_. Linear regression analysis of 1/T_1_ vs total iron concentration yielded the T_1,2_ relaxivity (r_1,2_).

#### Phantom MR imaging of Doxo-SPIO-micelles

To evaluate the clustering effect of SPIO in the micelle on MR sensitivity, we measured T_2_ relaxation rates (1/T_2_, s^− 1^) using a spin echo pulse sequence with the echo time (TR) = 6 s and the repetition time (TE) varied from 9 ms to 150 ms (*n* = 8) on a 4.7 T Varian INOVA scanner. Linear regression analysis of 1/T_2_ vs. total metal concentration yielded the T_2_ relaxivity (r_2_).

#### Cellular uptake behaviors of Doxo-SPIO-micelles

Cellular uptake of different formula of Doxo-SPIO-loaded micelle was evaluated through flow cytometry (FACS) analysis using H1299 non-small cell lung carcinoma cells. H1299 cells were seeded in 6-well plates (300,000 cells/well) in 2 mL DMEM with 10% FBS and incubated for 24 h, followed by co-incubation with Doxo-SPIO-loaded micelles at the Doxo concentration of 10 μg/mL for 2 h. The treated cells were washed three times with PBS before FACS analysis.

#### In vivo pharmacokinetic and biodistribution study

The enhanced thermodynamic stability of micelles through incorporation of SPIO was clearly distinguished from above experiments. In vivo pharmacokinetic stability of Doxo-SPIO-micelles was also identified in our experiment. Doxo-SPIO-micelles, Doxo-micelles and free Doxo were injected via a lateral tail vein into 20–22 g of BALB-C mice at a normalized dose (2.5 mg/kg as Doxo). 50 μL of blood was collected from the retroorbital plexus and immediately centrifuged at 2000 g for 2 min. Doxo concentration in plasma was measured using a microplate fluorescence reader (Spectra Max M5, Molecular devices, CA) after organic solvent extraction [24].

For the body distribution study, each organ was collected and washed with the PBS after sacrificing of mice treated with Doxo-SPIO-micelles, Doxo-micelles, and Doxo (control). For quantification of Doxo content in the kidneys, liver, lungs, spleen, muscle and heart after the injection, mice were euthanized for imaging. Organs were collected, washed with PBS. Organs collected from 3 of Doxo treated mice served as control. Fluorescence image was performed and analyzed with in vivo imaging system (FOBI, NeoScience Co. Republic of Korea).

### Statistical analysis

Quantitative data were presented as the mean ± standard deviation (stdev) and comparisons were carried out using t-text analysis. Statistical significances were described at (*P < 0.1, **P < 0.05, ***p < 0.01).

## Results

### Clustering of SPIO in the micelle core

#### Effect of Doxo-loading on hydrodynamic size of micelle

For the study, we systematically increased the weight ratio of SPIO while keeping the polymer to drug ratio the same. Figure 1B shows that the hydrodynamic diameter (D_H_) of the micelles increased with an increase of SPIO weight ratio. The SPIO-free empty micelles showed 24 ± 3 nm of diameter. The diameter increased to 80 ± 13, 100 ± 9 and 108 ± 8 nm, respectively, when SPIO were loaded in the micelles by weight ratio of 15, 30, and 50% (Fig. 1B). TEM image analysis was revealed the same results. Micelle diameter increased by loading amount of SPIO (Fig. 1C-F). This observation indicates that the size of micelle was affected efficiently from clustering of SPIO in the micelle core. Furthermore, iron content (%) of each Doxo-SPIO-micelle were estimated using an atomic absorption spectroscopy (Spectra AA240, Varian) for the study of theranostic property of Doxo-SPIO-micelles. As shown in Fig. S1, iron content increased to 11.8 ± 0.14, 15.7 ± 1.27 and 17.5 ± 0.99%, when the loading of SPIO was increased.

#### Clustering number of SPIO

The clustering number of SPIO was evaluated for the micelle core. Fig. S2 shows the relationship between individual clustering number of SPIO (N_ind_) and the resulting size of Doxo-SPIO-micelles. As demonstrated in Fig. 1 and Fig. S2 clearly shows that the size of the micelles increases in proportion to the clustering number of SPIO.

### SPIO clustering effect on thermodynamic stability of Doxo-SPIO-micelles

#### SPIO clustering effect on CMC of micelle

**Fig. 2:**
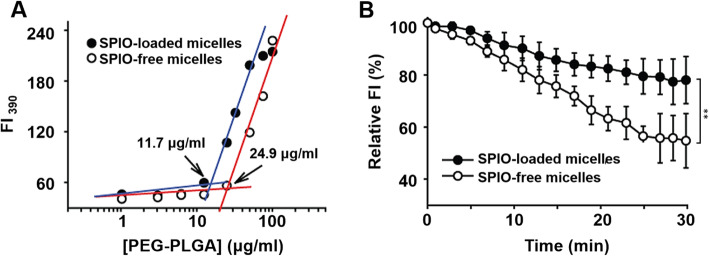
Effect of SPIO-clustering on thermodynamic stability of PEG-PLA polymeric micelles. (A) Effect of SPIO clustering on CMC of PEG-PLA (5 k–5 k) polymeric micelles. CMC was determined form fluorescence intensities of pyrene (l_**em**_ = 390 nm, l_**ex**_ = 333 nm) at different PEG-PLA concentrations. (B) Dissociation of SPIO-free micelles and SPIO-loaded micelles at the concentration of lower CMC (8 μg/mL) at 37 °C. Data are expressed as mean ± stdev, ** *p* < 0.05

The estimated the critical micelle concentration (CMC) of SPIO-loaded micelles and SPIO-free micelles were 11.7 μg/mL and 24.9 μg/mL, respectively (Fig. 2A). The micellization energy was calculated from the observed CMC by

1 $$ \varDelta {G}_o-{RTInX}_{cmc} $$

where R=gas constant (8.3143 J/K. mole), T=temperature (°K), Xcmc=concentration at CMC in molar fraction, and Go=micellization energy (kJ/mole) [25]. The micellization energy of SPIO loaded micelles and SPIO-free micelles were 29.3± 0.14 kJ/mole and -27.3±0.11 kJ/mole, respectively.

#### SPIO clustering effect on the dissociation of micelles

The concentration of SPIO-free micelles (24.9 μg/mL) at the turning point extrapolated from the graph matches. Interestingly, SPIO-micelles were displayed the well-accepted CMC value at 11.7 μg/mL (Fig. 2 A).

As shown in Fig. 2B, the initial fluorescence intensity of both micelles decreased gradually. The rate constant of dissociation for SPIO-free micelles was estimated as 0.55 ± 0.11 min^− 1^ at 37 °C. On the other hand, the dissociation rate constant for SPIO-loaded micelles was 0.26 ± 0.03 min^− 1^ at the same temperature.

### SPIO clustering effect on Doxo loading and release

#### SPIO clustering effect on the Doxo loading

**Fig. 3:**
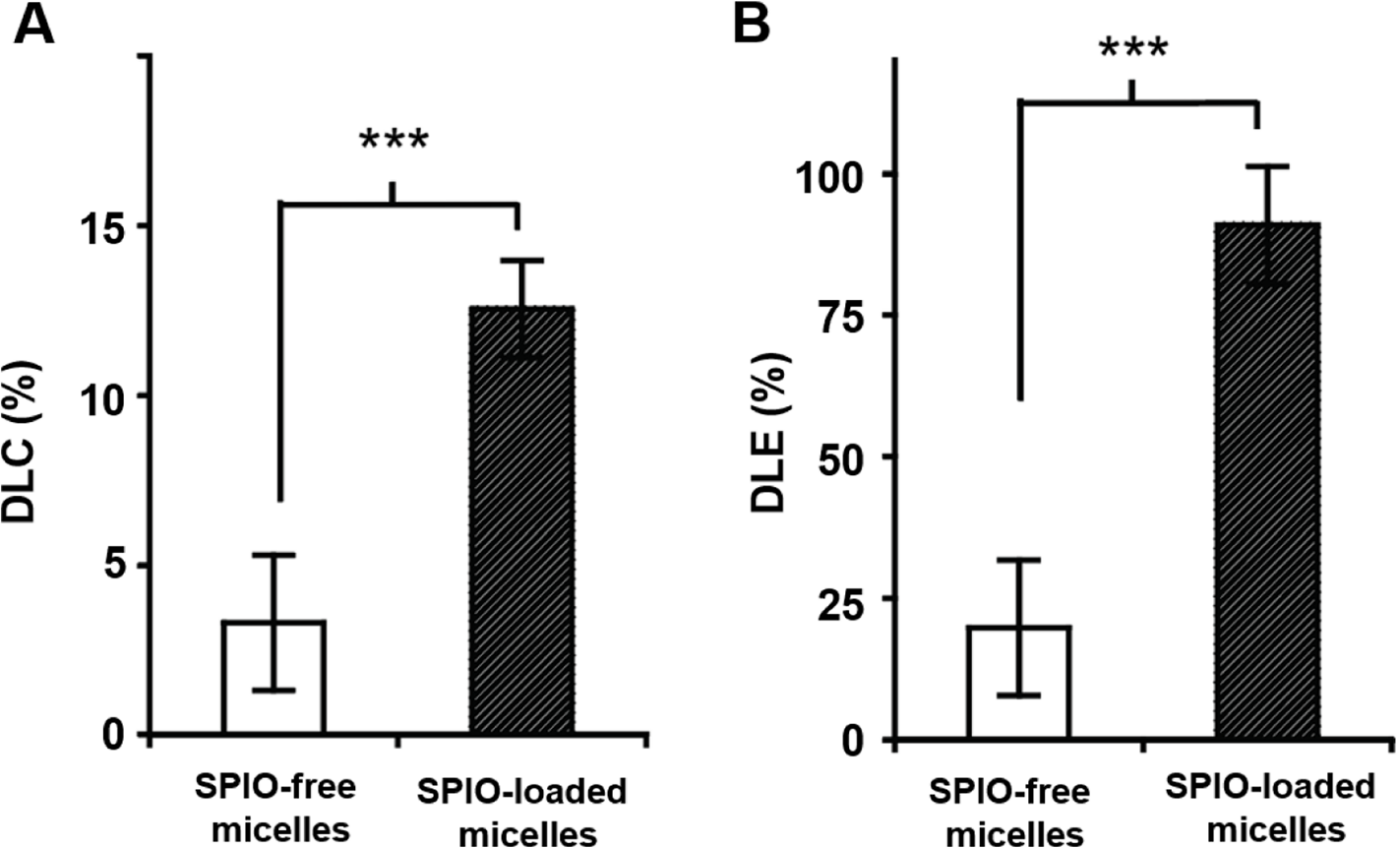
Effect of SPIO-clustering on drug loading of PEG-PLA polymeric micelles. (**A**) Drug loading content (DLC, %) and (**B**) drug loading efficiency (DLE, %) of SPIO-free and SPIO-loaded micelles (15% Doxo-loading). DLE (%) was calculated using the following equation: [estimated Doxo loading] / [theoretical Doxo loading] × 100. (**C**) Doxo loading content (%) of SPIO-loaded PEG-PLA micelles with mixing ratio of PEG-PLA and SPIO. Data are expressed as mean ± stdev, *** *p* < 0.01, ** *p* < 0.05

Incorporation of SPIO significantly increased the drug loading content (DLC) of Doxo, which was measured as the weight percentage of Doxo over the total weight of Doxo-SPIO-micelles. In SPIO-free micelles, the DLC was 3.3 ± 2.0%; while the DLC increased to 12.4% (*p* < 0.001, *n* = 4 when SPIO (15% wt) was co-encapsulated inside the micelles (Fig. 3A). Moreover, the encapsulation of SPIO also helped improve the drug loading efficiency (DLE), which was calculated as the weight percentage of encapsulated Doxo over the initial amount of drug. The SPIO-loaded micelles showed much higher DLE (90.9%) than the SPIO-free micelles (19.8 ± 2.0%, *p* < 0.001, n = 4, Fig. 3B).

#### Mechanism of increased drug loading on Doxo-SPIO-micelles

**Fig. 4:**
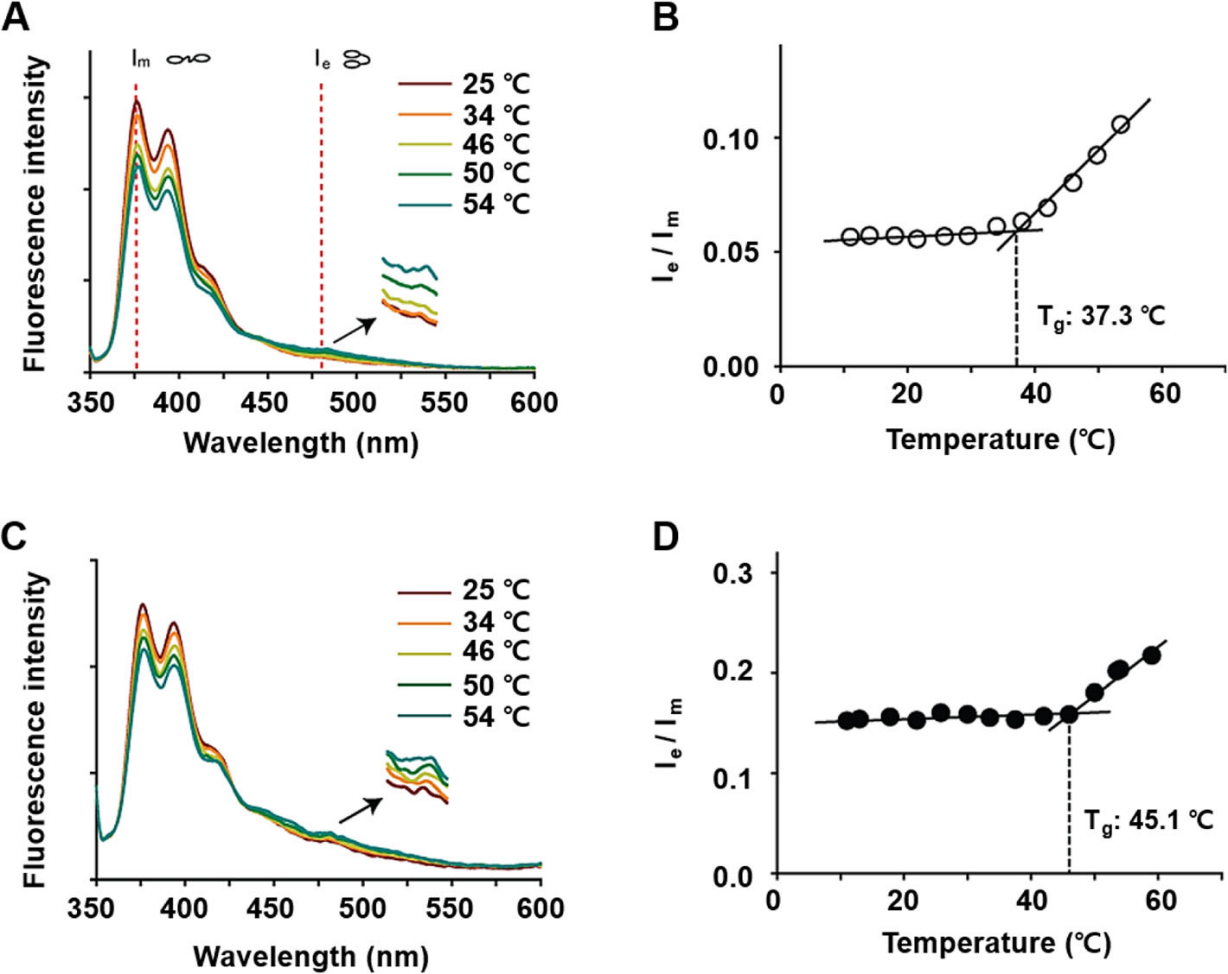
Effect of SPIO-clustering on thermodynamic stability of PEG-PLA polymeric micelles. Fluorescence emission spectra (l_ex=_399 nm) of 1,3-di(1,1′-pyrenyl)propane in SPIO-free micelles (**A**) and SPIO-loaded micelles (**C**). Fluorescence intensity ratio of excimer (l_em_ = 478 nm) to monomer (l_em_ = 377 nm) of 1,3-di (1,1′-pyrenyl)propane (*I*_e_ /*I*_m_) of SPIO-free micelles (B) and SPIO-micelles (**D**) at different temperatures

To elucidate the mechanism of increased drug loading, we used 1,3-(1,1′-dipyrenyl)-propane (DPP) dye to probe the microenvironment of micelle core. In this series of experiments, we immersed the SPIO-loaded or SPIO-free PEG-PLA micelles (we excluded Doxo from these micelles to avoid its fluorescence interference) in the DPP solution at 1.8 × 10^− 7^ M for 72 h under N_2_. The PEG-PLA concentrations were controlled at 100 μg/mL, well above CMC for each formulation (see CMC values below). The ratio of emission intensity of DPP excimer at 478 nm (I_e_) and monomer at 377 nm (I_m_) at an excitation of 333 nm was provided as a function of temperature (Fig. 4**)**. Figure 4A and C show SPIO-loaded micelles exert higher I_e_/I_m_ values than those of SPIO-free micelles at all range of temperatures. More specifically, the I_e_/I_m_ value of SPIO-free micelles at 37 °C was 0.07 compared to 0.19 for SPIO-loaded micelles. Generally, excimer formation is limited in a highly viscous environment, leading to relatively higher intensity of monomer (I_m_) and lower value of I_e_/ I_m_. Interestingly, micelles show a biphasic behavior with an inflection temperature at 37.3 °C (Fig. 4B), compared to 45.1 °C of SPIO-loaded micelles (Fig. 4D).

#### The effect of SPIO on the release of Doxo from micelles

**Fig. 5:**
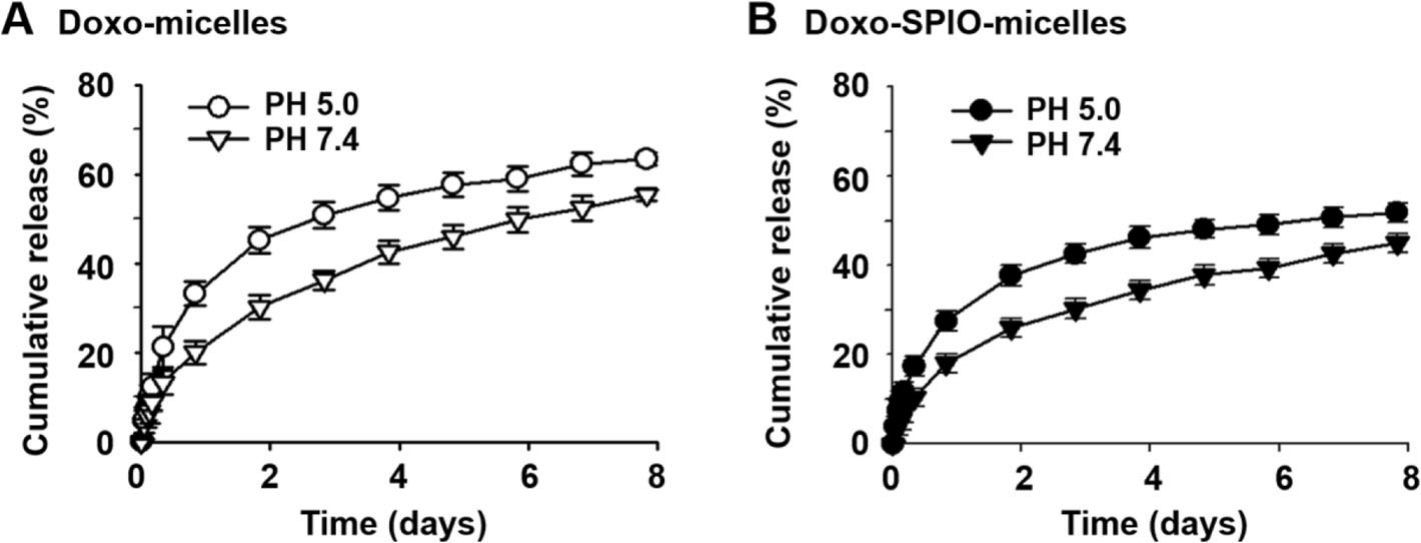
Effect of SPIO-clustering on drug release of PEG-PLA polymeric micelles. pH dependent release of Doxo from (**A**) SPIO free micelles (Doxo-micelles) and (**B**) Doxo-loaded micelles (Doxo-SPIO-micelles)

We also investigated the in vitro release profile of Doxo from Doxo-SPIO-micelles and Doxo-micelles (Fig. 5 and Fig. S3). First, we observed Doxo-SPIO-micelles exhibit a pH-dependent drug release that was originally observed in Doxo-micelles. And Doxo-SPIO-micelles showed more sustained release properties than Doxo-micelles. In addition, the release of Doxo from Doxo-SPIO-micelles was varied by the loading amount of SPIO (Fig. S3). The higher Doxo-loading showed the more precipitated release.

#### Effect of SPIO-clustering on MR sensitivity of Doxo-SPIO-micelles

**Fig. 6:**
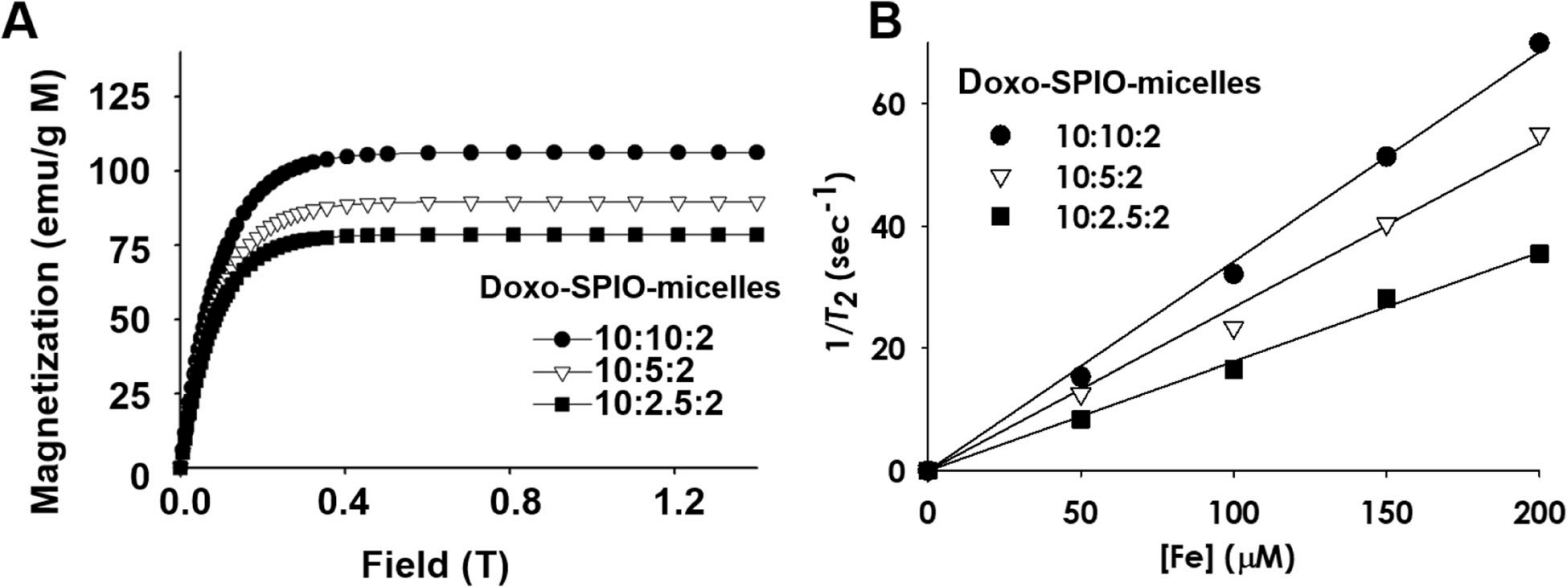
Effect of SPIO-clustering on MR sensitivity of PEG-PLA polymeric micelles. (A) Magnetization curves and (B) T_2_ relaxation rates (1/T_2_, sec^− 1^) of different formulations of Doxo-SPIO-micelles as a function of iron concentration. Doxo-SPIO-micelles were made from 10:2.5:2, 10:5:2 and 10:10:2 weight ratio of polymer:SPIO:Doxo

To help understand the MR properties, we measured the magnetization of Doxo-SPIO-loaded micelle using an alternating gradient magnetometer (AGM, Princeton Measurements) at room temperature and up to 0.4 T. (Fig. 6A). The values of magnetic moment at 0.4 T, where all the MRI measurements were conducted, reached the saturation value (M_sat_). The values of M_sat_ per unit metal mass were 78.6, 89.5 and 106.2 emu/g M for the micelle, made from 10:2.5:2, 10:5:2 and 10:10:2 ratio of components, respectively. The M_sat_ values are well correlated with those of r_2_, consistent with the hypothesis that higher magnetization would result in larger field in homogeneities surrounding SPIO and more effective T_2_ relaxation of water molecules (Fig. 6B).

Figure 6B shows that r_2_ values of Doxo-SPIO-micelles were further increased in PEG-PLA micelles where a cluster of SPIO was loaded. 7 nm Doxo-loaded micelle showed the increased r_2_ values (221.3, 290.5, and 319.8 mM^− 1^ s^− 1^), as the ratio of SPIO was increased (10:2.5:2, 10:5:2 and 10:10:2), respectively.

#### In vitro cellular uptake behavior of Doxo-SPIO-micelles

**Fig. 7:**
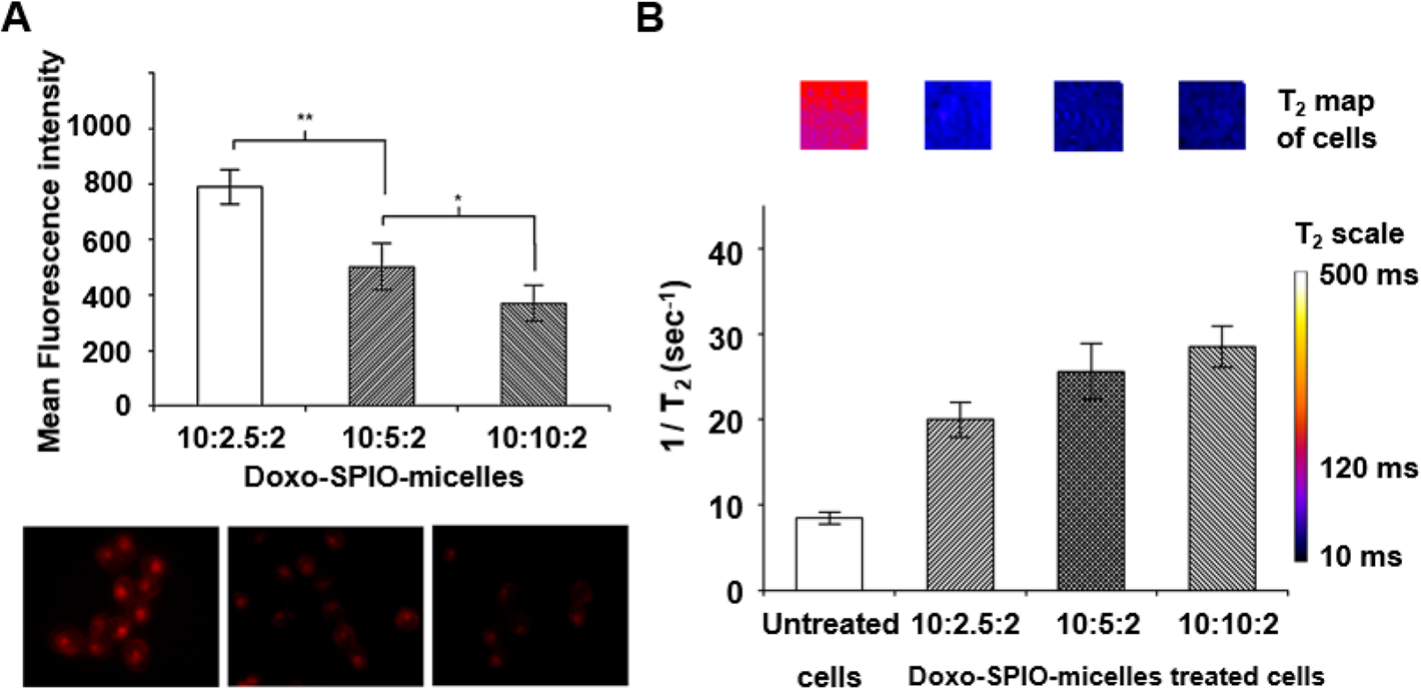
Effect of SPIO-clustering on cellular uptake behaviors of PEG-PLA polymeric micelles. (**A**) Mean fluorescence intensity of H1299 cells by flow cytometry (up). Fluorescence laser scanning microscopy of H1299 cells after 2 h of incubation (down). Doxo fluorescent images were acquired at λ_ex_ = 485 nm, λ_em_ = 595 nm. (**B**) T_2_ relaxation rates (1/T_2_, sec^− 1^) of the collected H2009 cells after treatment with Doxo-SPIO-micelles. The inset shows T_2_ maps of cell samples. Data are expressed as mean ± stdev, ** *p* < 0.05, * *p* < 0.1

The cellular uptake of each Doxo-SPIO-micelle was estimated by flow cytometry and confocal imaging analysis of cells. As shown in Fig. 7A and S4, the uptake decreased as the loading amount of SPIO increased. In the other hand, the MR sensitivity of cells after the uptake of Doxo-SPIO-micelles increased as the loading amount of SPIO increased (Fig. 7B and S4).

#### In vivo pharmacokinetics of Doxo-SPIO-micelles

**Fig. 8:**
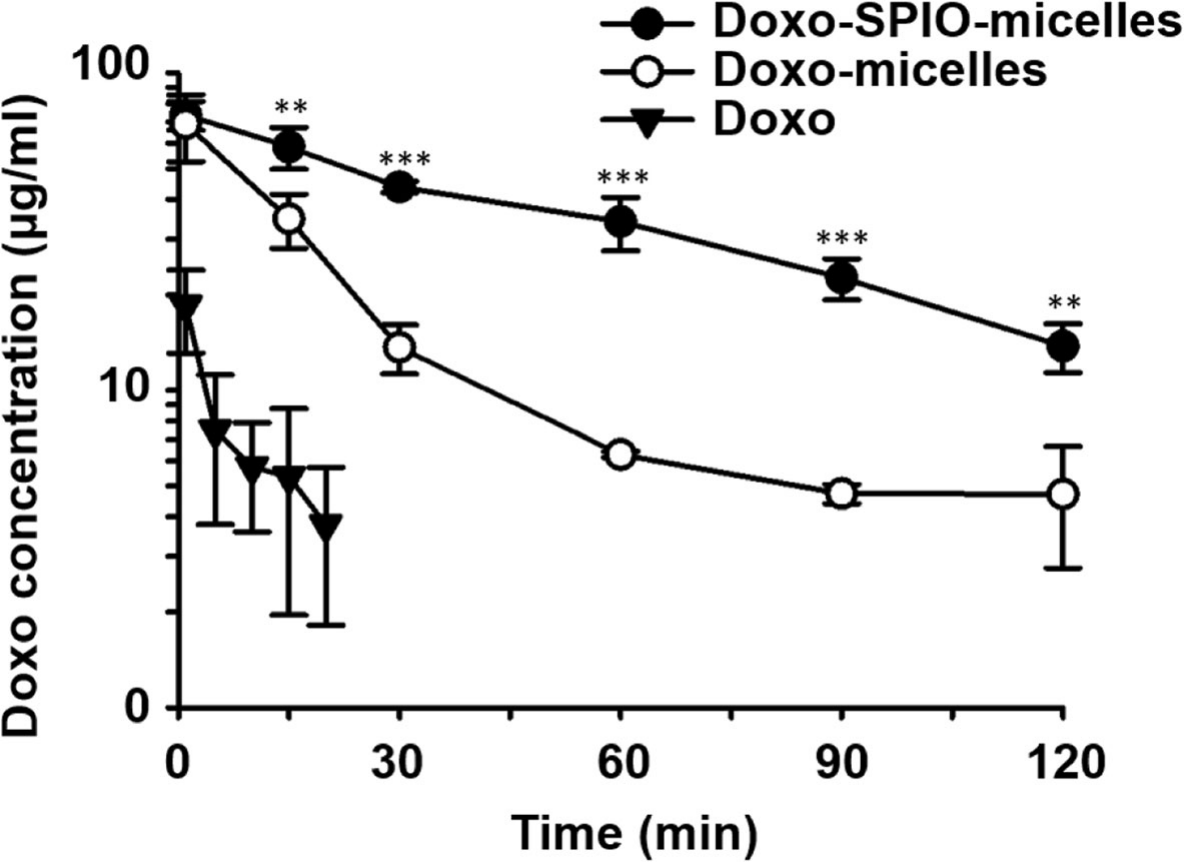
Effect of SPIO-clustering on pharmacokinetics of PEG-PLA polymeric micelles. Plasma concentration of Doxo was observed after i.v. injection of Doxo-SPIO-micelles, Doxo-micelles and free-Doxo at a 2.5 mg/kg Doxo dose. Statistical analysis of data was performed between Doxo-SPIO-micelles, Doxo-micelles (n = 5, data are expressed as mean ± stdev, *p < 0.1, **p < 0.05, ***p < 0.01)

**Fig. 9:**
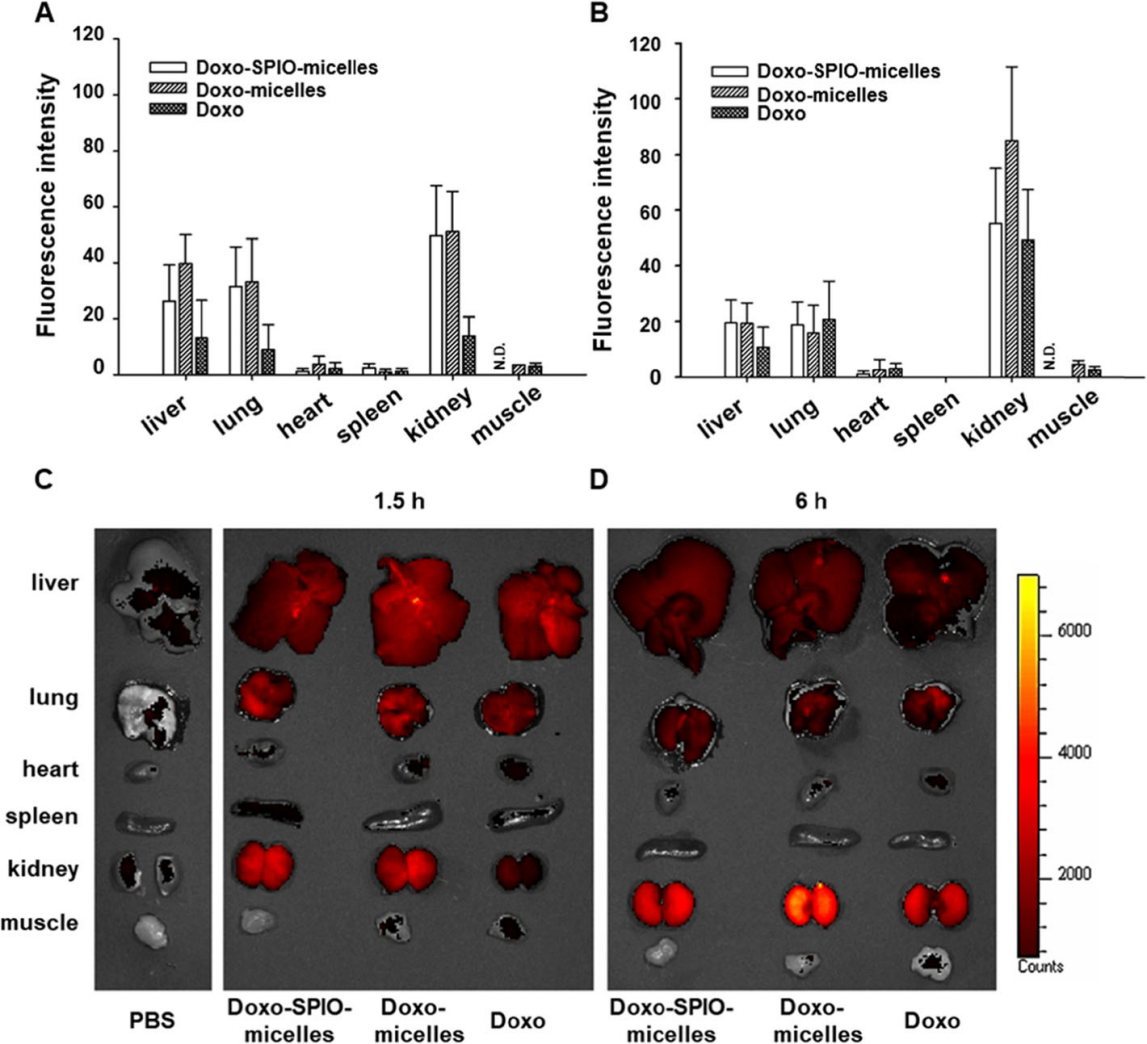
Effect of SPIO-clustering on organ distribution of PEG-PLA polymeric micelles. Fluorescence of each organ was observed after i.v. injection of Doxo-SPIO-micelles, Doxo-micelles and free-Doxo at a 2.5 mg/kg Doxo dose. (**A** and **B**) Mean fluorescence intensity of organs at 1.5 h and 6 h after the injection. (**C** and **D**) Photo-fluorescence images of organs at 1.5 h and 6 h after the injection

As shown in Fig. 8 and S5, Doxo-SPIO-micelles showed a sustained blood concentration profile, compared to Doxo-micelles and free Doxo. The Doxo-SPIO-micelles had an AUC of 3310.6 ± 449.7 μg·min/mL and a T_1/2_ of 50.5 ± 1.8 min, while Doxo-micelles had an AUC of 908.1 ± 85.5 μg·min/mL, and a T_1/2_ of 12.4 ± 0.6 min (Table 1). In addition, quantification of Doxo content in kidneys, liver, lungs, spleen, muscle, and heart were collected and from mice euthanized after experiments. Fluorescence images in each sample was obtained (Fig. 9C and D) and statistical graph of fluorescence intensity was determined (Fig. 9A and B). High concentration of Doxo were accumulated in liver, lung and kidney 6 h after an injection. Generally, Doxo-SPIO-micelles showed higher accumulation on the organs at initial phase of distribution. For the subsequent study, we observed the effect of the amount of Doxo-loading on pharmacokinetics of Doxo-SPIO-micelles. As shown in the Fig. S5, Doxo-SPIO-micelles with higher SPIO-loading showed more rapid decline of blood concentration (Fig. S5 and Table S1). It may be related with the difference in cellular uptake efficiency by SPIO-loading amount (Fig. 7). However, we could not observe the large difference of organ fluorescence intensity between treatments by SPIO-loading. So, Prussian blue staining was carrying out to determine the accumulation of Doxo-SPIO-micelles on lung, liver, and kidney after an intravenous injection of Doxo-SPIO-micelles with different Doxo-loading, as shown in Fig. S6–8.

**Table 1:** Pharmacokinetic parameters after intravenous injection of free Doxo, Doxo-micelles, and Doxo-SPIO-micelles against mice at a dose of Doxo 2.5 mg/kg (*n* = 5, mean ± stdev)

	Free Doxo	Doxo-micelles	Doxo-SPIO-micelles
AUC (μg·min/mL)	44.4 ± 4.6	908 ± 86	3310.6 ± 449.7
t_1/2_ (min)	2.1 ± 0.6	12.4 ± 0.6	50.5 ± 1.8
CL (mL/min/kg)*	56.2 ± 10.9	2.7 ± 0.2	0.76 ± 0.1
MRT (min)**	3.0 ± 0.8	17.9 ± 0.9	72.9 ± 2.6
V_ss_ (mL)	172.9 ± 27.5	49.0 ± 7.3	55.4 ± 5.5

* CL; drug clearance, ** MRT: mean residence time

## Discussion

In this work, Doxo-loading polymeric micelles and SPIO-free polymeric micelles were synthesized to confirm the effect of Doxo-loading on hydrodynamic size and stability of micelle. Here, we observed the clustering of SPIO in the micelle core affected the size of micelle due to hydrophobic SPIO and micellization energy. The negative values of the micellization energy indicate that the micelle formation is spontaneous as it is a thermodynamically favorable process. In addition, SPIO induce stronger hydrophobic interaction, formed kinetically more stable micelles, and would maintain their structural integrity for longer time even below the CMC. According to the excellent stability of SPIO-loading micelles, effect of SPIO improved DLE on drug loading and release. Further study, DPP is exposed in viscous or glassy environment, excimer form of DPP significantly decreases, resulting in a lower value of I_e_/I_m_ [26]. We consider this reflects the glass transition temperature (T_g_) of the PLA micelle core in aqueous solution. At the temperatures above the T_g_, the micelle core becomes more fluidic, therefore facilitates the formation of excimer over monomer. This value (37.3 °C) is consistent with the T_g_ value as determined by Kataoka and coworkers by the ^1^H NMR experiments [27]. This biphasic behavior was absent in SPIO-loaded micelles. In addition, the I_e_/I_m_ values were much lower for SPIO-free micelles than SPIO-loaded micelles at all temperatures. More specifically at 37 °C, the I_e_/I_m_ value for SPIO-free micelles was 0.07 compared to 0.19 for SPIO-loaded micelles. These data suggest that incorporation of SPIO considerably increases the fluidity and flexibility in the micelle core, which may reflect the plasticizing effect of the unsaturated hydrocarbon chains of oleic acids from SPIO surface [28]. Together with the increase of the micelle core volume as the result of Doxo-loading, these two factors may be the primary contributing factor for the dramatic increase in drug loading content and efficiency.

In addition, our study indicated that release of Doxo from SPION-micelles was sustained and pH-sensitive Doxo release, that is suggesting a stronger Doxo-micelle core interaction compared to the interaction in SPIO-free Doxo-micelles. However, both micelle formulations showed pH-sensitive release properties, which is beneficial for Doxo accumulation to the acidic tumor environment [14, 17, 29]. As we know, SPIO, that serve as an MRI candidate, affected the MR property, and the highest increase of r_2_ values was observed in 7 nm Doxo-loaded micelles (10:10:2) with the highest clustering tendency in TEM images. It suggested that clustering enhanced the MR sensitivity. This also suggests polymeric micelle can be the most profit nano design to get highly sufficient MR sensitivity in vivo.

Generally, Surface chemistry of nanoparticles such as surface charge, targeting moiety and lipophilicity highly affects the cellular uptake of nanoparticles [30–32]. In this study, surface chemistry is not so proper to explain the observed different uptake efficiency, because all Doxo-SPIO-micelles have the same surface chemistry with PEG (5 k) of corona layer.

Particle size also has been regarded as potential factor affecting cellular uptake. Desai et al. proved PLGA microparticles with different size of 10 μm, 1 μm and 0.1 μm showed clearly different cellular uptake efficiency [33]. The 0.1 μm diameter particles had 2.5- and 6-folds higher uptake than 1 and 10 μm diameters of microparticles, respectively. However, for the nanoparticles in range of 10 to 100 nm, the effect of different size on the cellular uptake remains poorly understood. Recently, Chithrani et al. proved 50 nm of gold nanoparticles had a much higher uptake efficiency compared to the other size (e.g. 20 or 100 nm) of gold nanoparticles [34]. The other study also proved the 107 nm dextran crosslinked SPIO exhibited the highest uptake efficiency by T cells, compared to 33.4 and 52.5 nm of dextran crosslinked SPIO [35]. These observations imply the cellular uptake of nanoparticles (around 100 nm) is not always dependent on the size. For the polymeric micelles, the efficient way to control the micelle size by 10 to 20 nm units like inorganic nanoparticles is not fully established yet. Consequently, the size dependency on the cellular uptake of micelles is still unambiguous. In this study, the cellular uptake of Doxo-SPIO-micelles proved to be dependent on the amount of SPIO in micelle core as well as micelle size. Thus, we hypothesize that SPIO possibly change “the rigidity of micelles” that may affect trafficking process of micelle through cell membrane. For the proof of this hypothesis, the more intensive studies are required on this stage.

In vivo study, a sustained blood concentration and high intensity of Doxo on the kidney, lung, and liver were observed at Doxo-SPIO-micelles, whereas Doxo-micelles and Doxo showed more rapid clearances evidently. Interestingly, Figs. 8, 9, and S5 suggested higher loading of SPIO on micelles precipitated the rapid elimination of Doxo-SPIO-micelles. Prussian blue staining, as displayed Fig. S6, S7 and S8, also proved more rapid elimination in higher SPIO-loading micelles.

In vivo theranostic functions of SPIOs have been thoroughly studied for decades, involved enhancing MRI images, hyperthermia, cytotoxic effect, and tumor-targeted therapy. Interestingly, scientists have randomly used hydrophobic SPIOs and hydrophilic SPIOs for the studies. And some studies tried the transformation of hydrophobic iron oxide nanoparticles (INOPs) by ligand exchange to enhance the water-solubility [36, 37]. Hydrophobic iron oxide cluster co-polymer micelle exhibited specific targeted delivery and diagnostic functions [38]. The study indicated that degradation of INOPs and interaction with ferritin through phagocytic, metabolic and degradative processes by macrophages of liver and spleen and blood circulation [39, 40]. And INOPs were accumulated highly in spleen and liver. In additional, Barbara Freund and his research group performed in vivo quantification studies of ^59^Fe-radiolabel monodisperse SPIO, suggesting that most of the labeled oleic acid shell is separated from the iron oxide cores [41]. As we know, hydrophobic components mostly stay in blood compartment and eliminated by liver. Furthermore, iron ion distributed in the liver and spleen after 1 day and 4 weeks after injection of phospholipid-modification SPIO. Those results suggests that hydrophobic SPIOs are stored in liver and spleen and display slow rate of degradation and clearance. And degradation products may contribute to subclinical toxicity [42]. However, our study proved Doxo-SPIO-micelles showed more sustained release of Doxo and delayed clearance of micelles by itself.

## Conclusion

In summary, we have reported the effects of co-encapsulation of SPIO with Doxo into polymeric micelles on the physical and biological properties of the micelles in vitro and in vivo. Clustering of SPIO inside Doxo-micelles significantly increased drug loading content and drug loading efficiency. SPIO improved the thermodynamic stability of the micelles by decreasing their CMC and the kinetic stability. Doxo-SPIO-micelles also showed prolonged circulation half-life in animals, and superb in vivo stability as shown by TEM and MRI. The improved properties of the micelles will allow for the development of integrated nanomedicine with high drug loading contents, prolong the blood half-life, and excellent in vitro and in vivo stabilities for future therapeutic and diagnostic applications.

## Supplementary Information

**Additional file 1: Fig. S1.** Iron content of Doxo-SPIO-micelles. The applied weight ratios of PEG-PLA:SPIO:Doxo for the preparation of each micelles were 10:2.5:2, 10:5:2, and 10:10:2, respectively. The iron content (%) was estimated via atomic absorption spectroscopy (Spectra AA240, Varian). **Fig. S2.** The relationship between individual clustering number of SPIO (N _ind_) and the resulting size of SPIO-loaded micelles. **Fig. S3.** Effect of SPIO-loading amount on drug release of PEG-PLA polymeric micelles. **Fig. S4.** Cellular uptake of different formula of D oxo-SPIO-micelles micelle in H1299 non-small cell lung carcinoma cells. **Fig. S5.** Effect of SPIO-loading amount on pharmacokinetics of PEG-PLA polymeric micelles. Plasma concentration of Doxo was observed after i.v. injection of Doxo-SPIO-micelles with different SPIO loading ratios (*n* = 3, data are expressed as mean ± stdev). **Table S1.** Pharmacokinetic parameters after intravenous injection of SPIO-Doxo-micelles with different SPIO loading ratios against mice at a dose of 2.5 mg Doxo·kg^− 1^ (n = 3, mean ± stdev). **Fig. S6.** Accumulation of Doxo-SPIO-micelles on lung, liver, and kidney after an intravenous injection of Doxo-SPIO-micelles (10:2.5:2). Organs were recovered 30 and 60 min after an injection. H&E and Prussian blue staining were performed to observe the accumulation of SPIO on each organ. **Fig. S7.** Accumulation of Doxo-SPIO-micelles on lung, liver, and kidney after an intravenous injection of Doxo-SPIO-micelles (10:5:2). Organs were recovered 30 and 60 min after an injection. H&E and Prussian blue staining were performed to observe the accumulation of SPIO on each organ. **Fig. S8.** Accumulation of Doxo-SPIO-micelles on lung, liver, and kidney after an intravenous injection of Doxo-SPIO-micelles (10:10:2). Organs were recovered 30 and 60 min after an injection. H&E and Prussian blue staining were performed to observe the accumulation of SPIO on each organ.

## Data Availability

The datasets and materials used and/or analyzed during the current study are available from the corresponding author on reasonable request.

## Abbreviations

SPIO: Superparamagnetic iron oxide nanoparticles
Doxo: doxorubicin
PEG-PLA: poly(ethylene glycol)-co-poly(D,L-lactide)
AUC: area under curve
MRI: magnetic resonance imaging
Doxo-SPIO-micelles: SPIO and Doxo loaded polymeric micelles
DLC: drug loading content
CMC: critical micelle concentration
DLE: drug loading efficiency
TEM: transmission electron microscopy
I_e_: 1,3-(1,1′-dipyrenyl)-propane (DPP) emission intensity of DPP excimer at 478 nm
I_m_: emission intensity of DPP monomer at 377 nm
TE: the echo time
TR: the repetition time
CL: drug clearance
MRT: mean residence time
