# Supplementary material for: Enhanced thermodynamic, pharmacokinetic and theranostic properties of polymeric micelles via hydrophobic core-clustering of superparamagnetic iron oxide nanoparticles
Source: Biomater Res. 2022 Mar 7;26:8. doi: 10.1186/s40824-022-00255-9 (PMC8900364; doi:10.1186/s40824-022-00255-9)
Supplement: Supplementary file 1 — Additional file 1: Fig. S1. Iron content of Doxo-SPIO-micelles. The applied weight ratios of PEG-PLA:SPIO:Doxo for the preparation of each micelles were 10:2.5:2, 10:5:2, and 10:10:2, respectively. The iron content (%) was estimated via atomic absorption spectroscopy (Spectra AA240, Varian). Fig. S2. The relationship between individual clustering number of SPIO (N ind) and the resulting size of SPIO-loaded micelles. Fig. S3. Effect of SPIO-loading amount on drug release of PEG-PLA polymeric micelles. Fig. S4. Cellular uptake of different formula of D oxo-SPIO-micelles micelle in H1299 non-small cell lung carcinoma cells. Fig. S5. Effect of SPIO-loading amount on pharmacokinetics of PEG-PLA polymeric micelles. Plasma concentration of Doxo was observed after i.v. injection of Doxo-SPIO-micelles with different SPIO loading ratios (n = 3, data are expressed as mean ± stdev). Table S1. Pharmacokinetic parameters after intravenous injection of SPIO-Doxo-micelles with different SPIO loading ratios against mice at a dose of 2.5 mg Doxo·kg− 1 (n = 3, mean ± stdev). Fig. S6. Accumulation of Doxo-SPIO-micelles on lung, liver, and kidney after an intravenous injection of Doxo-SPIO-micelles (10:2.5:2). Organs were recovered 30 and 60 min after an injection. H&E and Prussian blue staining were performed to observe the accumulation of SPIO on each organ. Fig. S7. Accumulation of Doxo-SPIO-micelles on lung, liver, and kidney after an intravenous injection of Doxo-SPIO-micelles (10:5:2). Organs were recovered 30 and 60 min after an injection. H&E and Prussian blue staining were performed to observe the accumulation of SPIO on each organ. Fig. S8. Accumulation of Doxo-SPIO-micelles on lung, liver, and kidney after an intravenous injection of Doxo-SPIO-micelles (10:10:2). Organs were recovered 30 and 60 min after an injection. H&E and Prussian blue staining were performed to observe the accumulation of SPIO on each organ. [file 40824_2022_255_MOESM1_ESM.docx]

**Supplementary Information**

**Enhanced thermodynamic, pharmacokinetic and theranostic properties of polymeric micelles via hydrophobic core-clustering iron oxide nanoparticles**

Yixin Jiang ^1,2,‡^, Junghan Lee ^1,2,‡^, Jin-Myung Seo^1^, Enkhzaya Davaa^1^, Kyung-Ju Shin^1^, and Su-Geun Yang^1,2*^

^1^Department of Biomedical Science, BK21 FOUR Program in Biomedical Science and Engineering, Inha University College of Medicine, Incheon 22212, Republic of Korea

^2^Inha Institute of Aerospace Medicine, Inha University College of Medicine, Incheon 22332, Republic of Korea

*Corresponding author: E-mail: [sugeun.yang@inha.ac.kr](mailto:sugeun.yang@inha.ac.kr)

^‡^ Yixin Jiang and Junghan Lee contributed equally to this work.

**1. Iron content of Doxo-SPIO-micelles.**


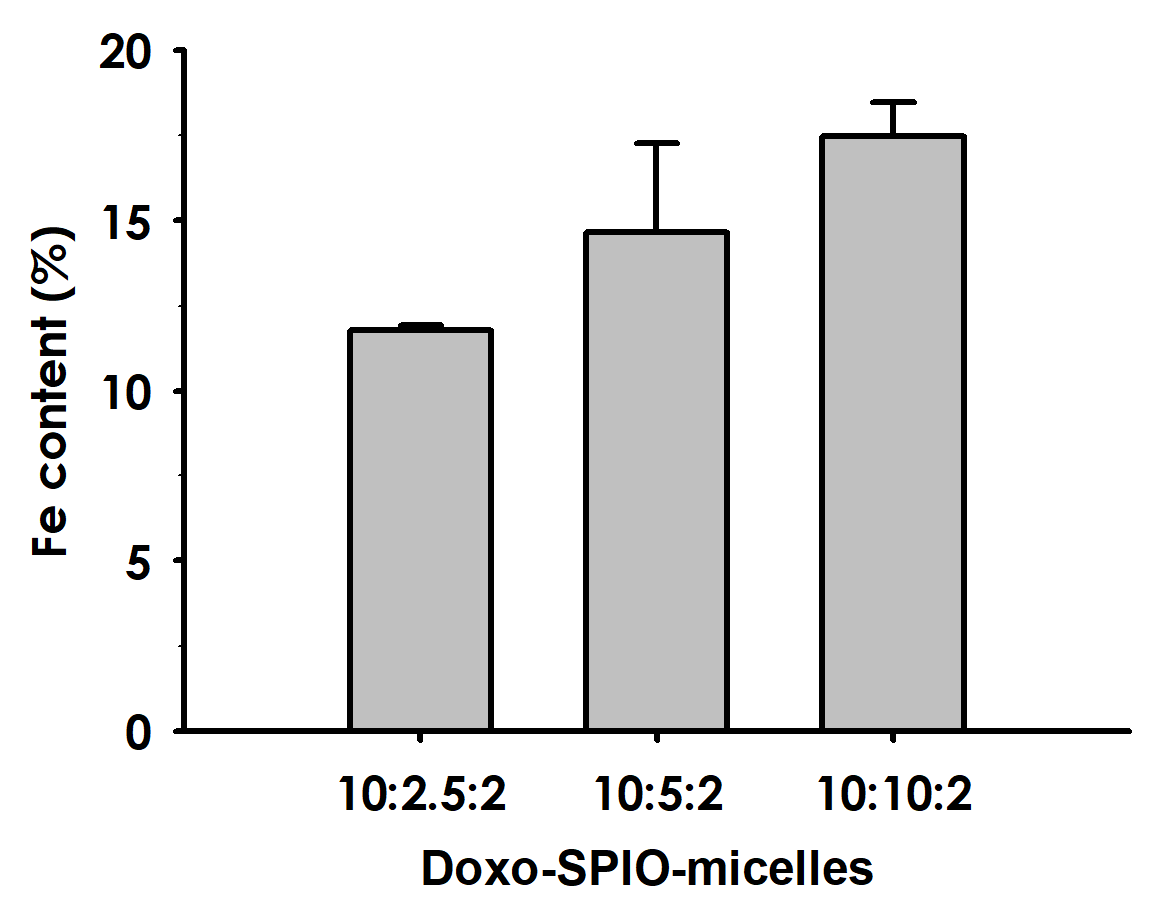


**Fig. S1** Iron content of Doxo-SPIO-micelles. The applied weight ratios of PEG-PLA:SPIO:Doxo for the preparation of each micelles were 10:2.5:2, 10:5:2, and 10:10:2, respectively. The iron content (%) was estimated via atomic absorption spectroscopy (Spectra AA240, Varian).

**2. Clustering number of SPIO**

To evaluate the clustering effect of SPIO on MR sensitivity, single SPIO micelle was prepared following the reported method and MR sensitivity was measured in same way. In brief, DSPE-PEG copolymer was used to produce single SPIO loaded micelles due to its short hydrophobic chains. Single SPIO-loaded DSPE-PEG micelles were formed by the rehydration of a blended film of SPIO nanoparticles and DSPE-PEG with pH 7.4 HEPES buffer at 70 °C. An excess amount of DSPE-PEG (weight ratio to SPIO= 20:1) was used to ensure the formation of single SPIO micelles.


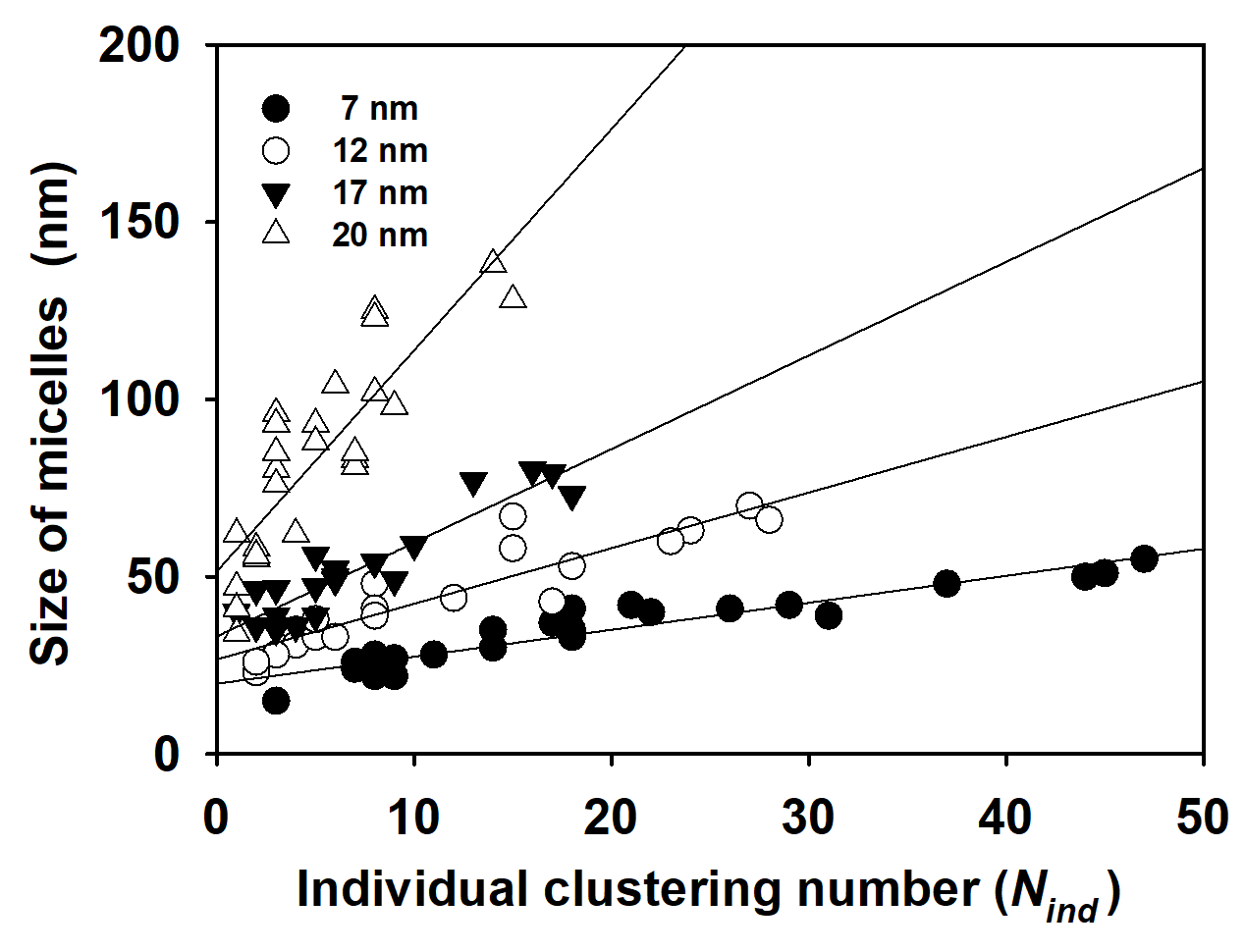


**Fig. S2** The relationship between individual clustering number of SPIO (N _ind_) and the resulting size of SPIO-loaded micelles.

**3. The effect of SPIO loading amount on the release of Doxo**


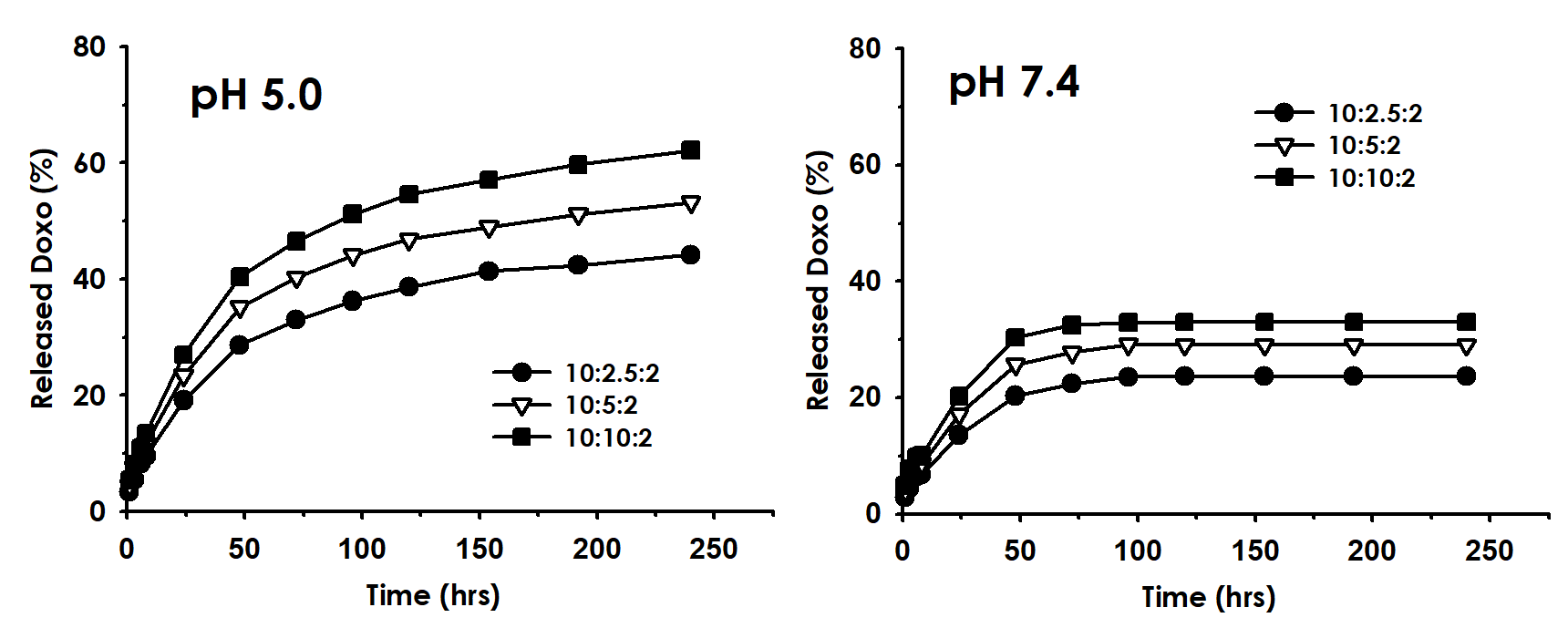


**Fig. S3** Effect of SPIO-loading amount on drug release of PEG-PLA polymeric micelles.

**4. Cellular uptake of SPIO-Doxo-loaded micelle**

Cellular uptake of different formula of SPIO-Doxo-loaded micelle was evaluated through flow cytometry analysis using H1299 non-small cell lung carcinoma cells. H1299 cells were seeded in 6-well plates (300,000 cells/well) in 2 ml DMEM with 10% FBS and incubated for 24 hrs, followed by co-incubation with SPIO-Doxo-loaded micelles at the Doxo concentration of 10 µg/ml for 2hrs.

After the treatment, the cells were washed three times with PBS, detached by trypsinization, centrifuged and dispersed again in PBS. The dispersed cells were introduced to flow cytometer (Cell Lab Qunta^TM^ SC, Beckman Coulter, CA).

**Fig. S4** Cellular uptake of different formula of D oxo-SPIO-micelles micelle in H1299 non-small cell lung carcinoma cells.

**5. Pharmacokinetics of SPIO-Doxo-loaded micelle with different SPIO loading ratio**


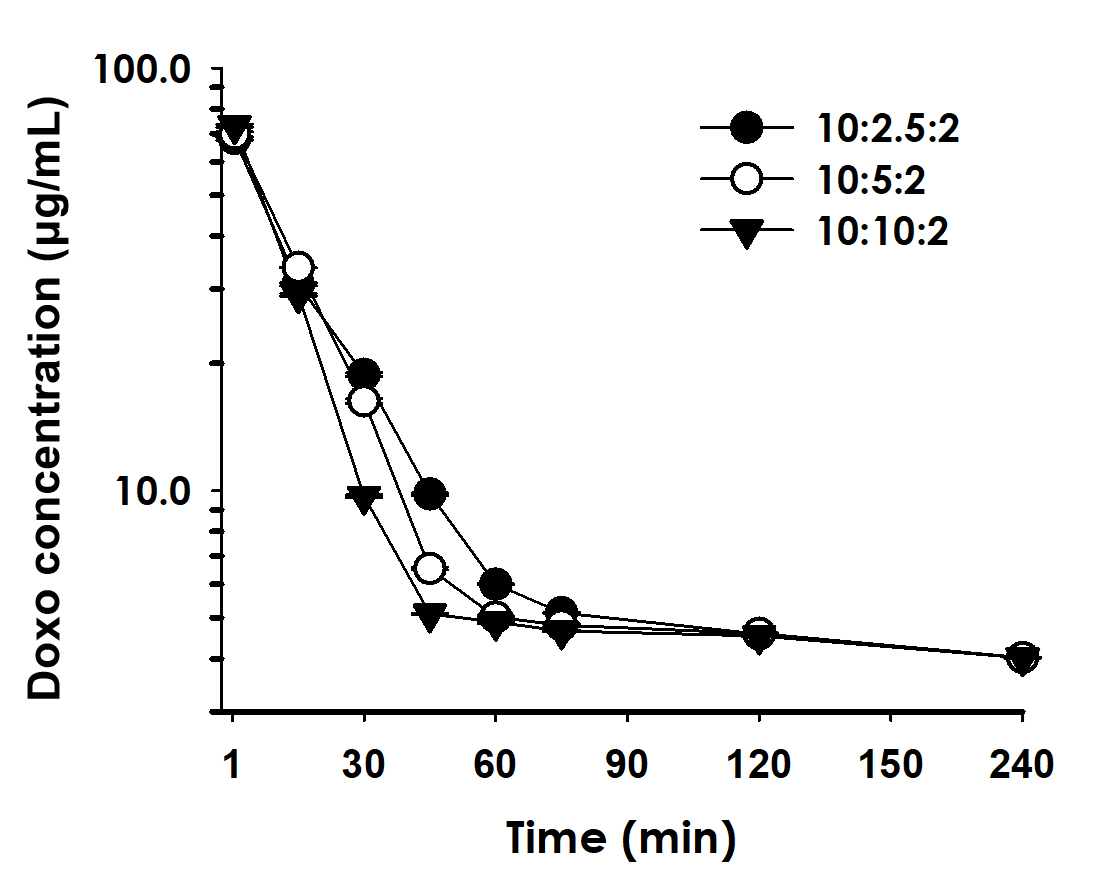


**Fig. S5** Effect of SPIO-loading amount on pharmacokinetics of PEG-PLA polymeric micelles. Plasma concentration of Doxo was observed after i.v. injection of Doxo-SPIO-micelles with different SPIO loading ratios (n=3, data are expressed as mean±stdev).

**Table S1** Pharmacokinetic parameters after intravenous injection of SPIO-Doxo-micelles with different SPIO loading ratios against mice at a dose of 2.5 mg Doxo·kg^-1^ (n=3, mean ± stdev).

|  | Doxo-SPIO-micelles (PEG-PLA:SPIO:Doxo) | | |
| --- | --- | --- | --- |
|  | 10:2.5:2 | 10:5:2 | 10:10:2 |
| AUC (μg·min/mL) | 3962.2 ± 19.1 | 3852.8 ± 15.9 | 3503.6 ± 13.7 |
| t_1/2_ (min)* | 32.7 ± 0.1 | 26.3 ± 0.2 | 22.7 ± 0.1 |
| CL (mL/min/kg) | 0.63 ± 0.003 | 0.65 ± 0.003 | 0.71 ± 0.006 |

* Plasma half-life of Doxo was estimated from first decline phase of plasma profile.


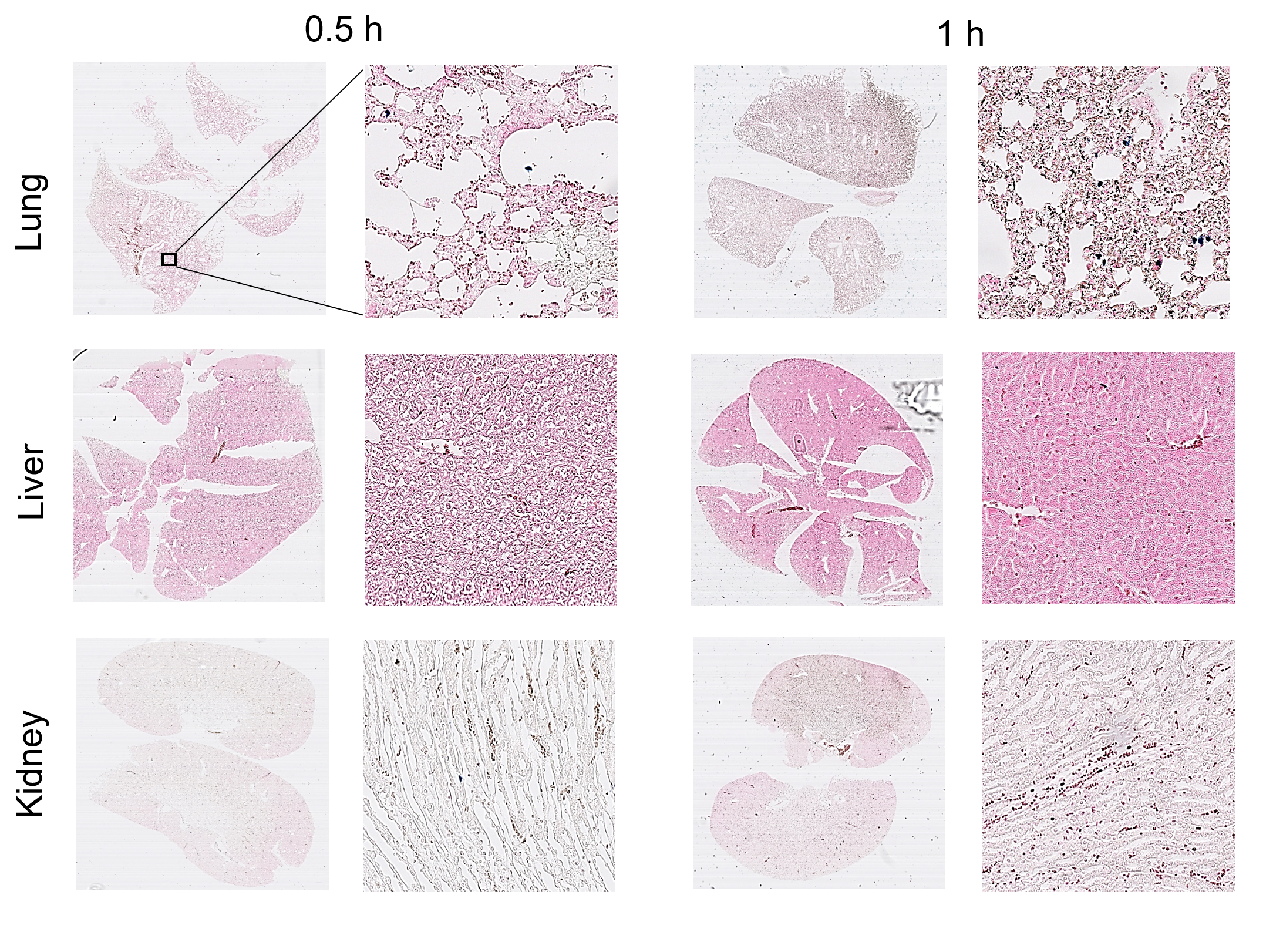


**Fig. S6** Accumulation of Doxo-SPIO-micelles on lung, liver, and kidney after an intravenous injection of Doxo-SPIO-micelles (10:2.5:2). Organs were recovered 30 and 60 minutes after an injection. H&E and Prussian blue staining were performed to observe the accumulation of SPIO on each organ.


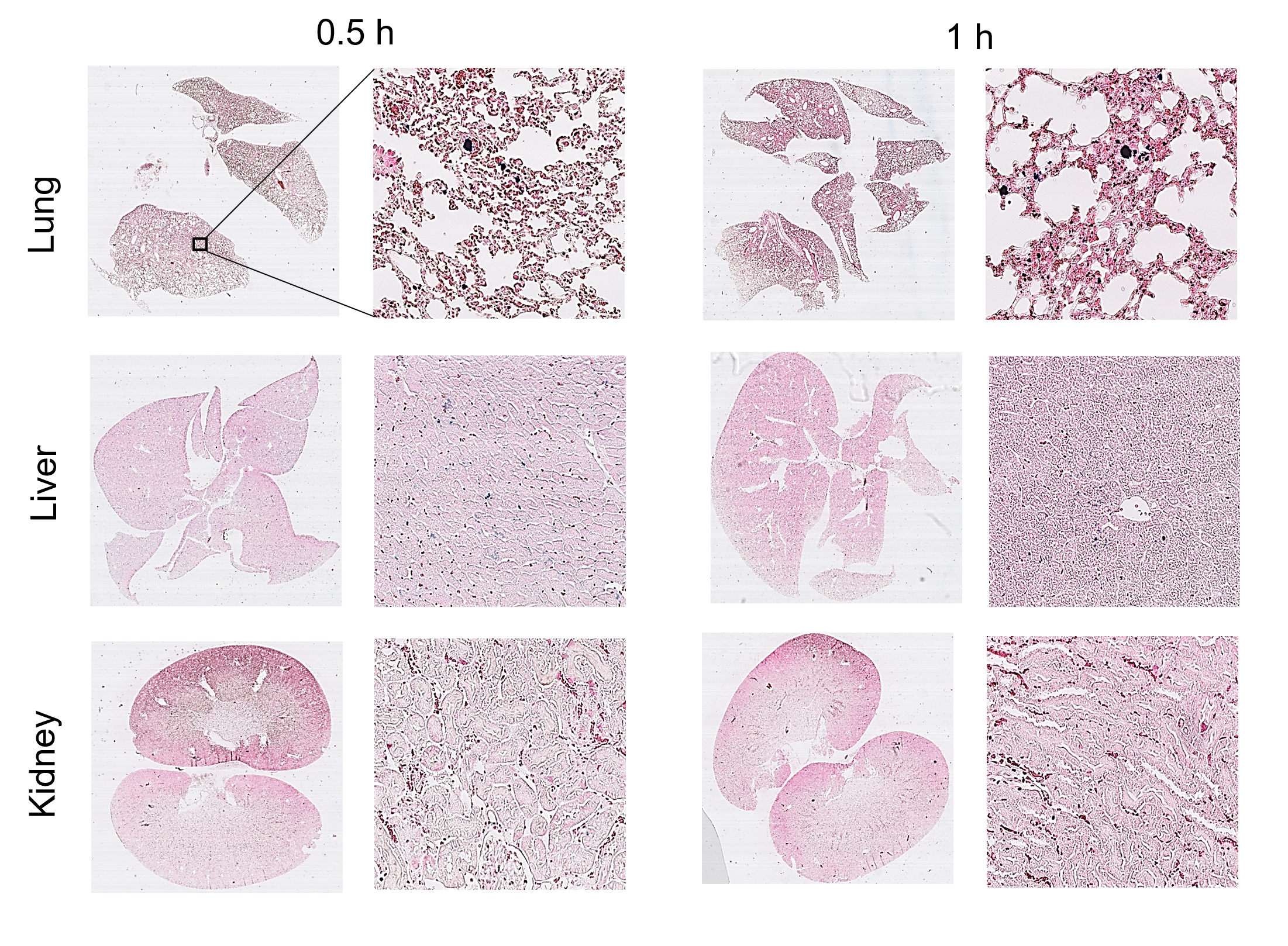


**Fig. S7** Accumulation of Doxo-SPIO-micelles on lung, liver, and kidney after an intravenous injection of Doxo-SPIO-micelles (10:5:2). Organs were recovered 30 and 60 minutes after an injection. H&E and Prussian blue staining were performed to observe the accumulation of SPIO on each organ.


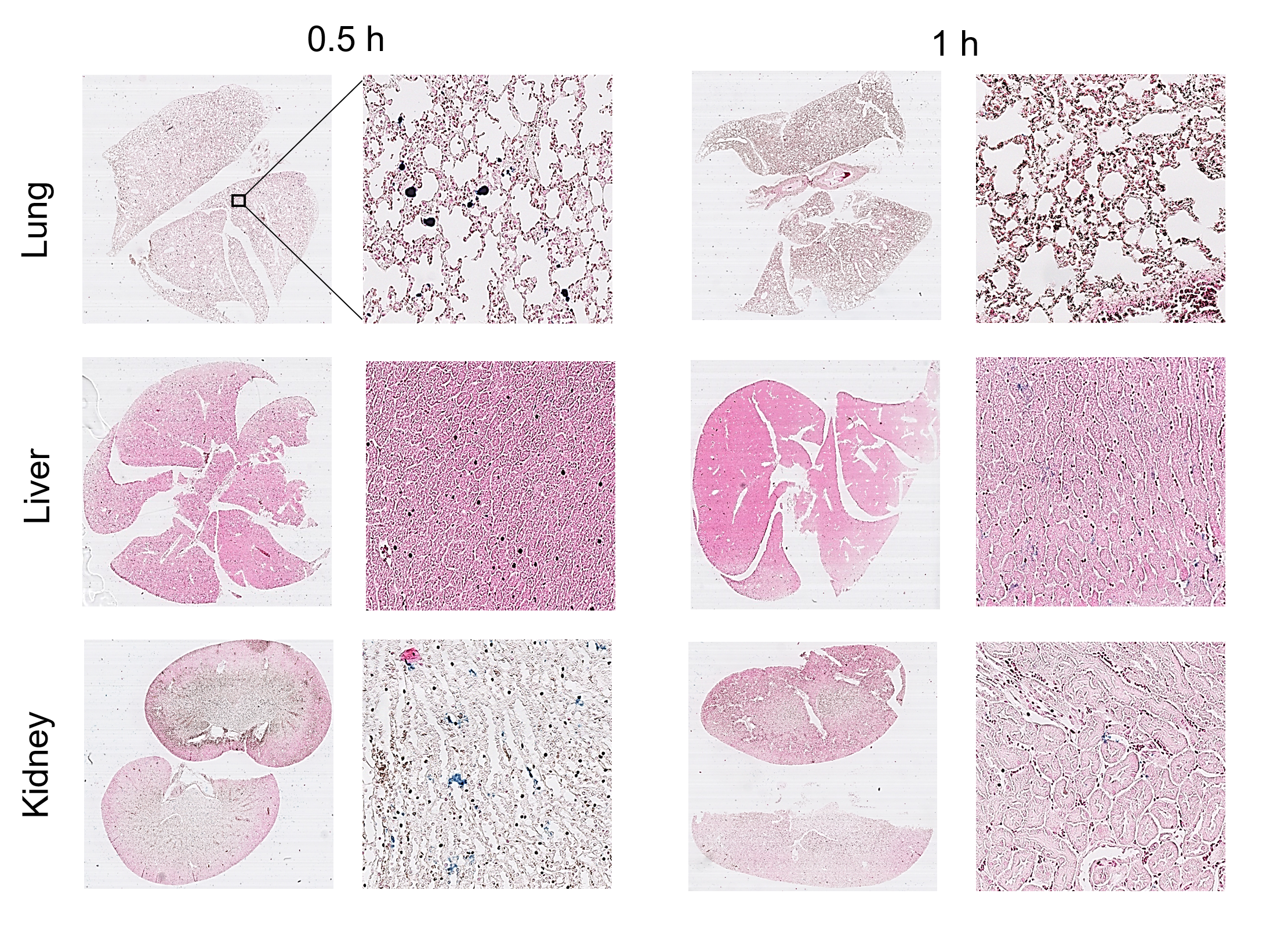


**Fig. S8** Accumulation of Doxo-SPIO-micelles on lung, liver, and kidney after an intravenous injection of Doxo-SPIO-micelles (10:10:2). Organs were recovered 30 and 60 minutes after an injection. H&E and Prussian blue staining were performed to observe the accumulation of SPIO on each organ.
